# Urinary CD8^+ ^T-cell counts discriminate between active and inactive lupus nephritis

**DOI:** 10.1186/ar4189

**Published:** 2013-02-27

**Authors:** Sebastian Dolff, Wayel H Abdulahad, Suzanne Arends, Marcory CRF van Dijk, Pieter C Limburg, Cees GM Kallenberg, Marc Bijl

**Affiliations:** 1 Department of Rheumatology and Clinical Immunology, University Medical Center Groningen, Hanzeplein 1, 9713 GZ Groningen, The Netherlands; 2 Department of Nephrology, University Hospital Essen, University Duisburg-Essen, Hufelandstr. 55, 45122 Essen, Germany; 3 Department of Pathology and Medical Biology, University Medical Center Groningen, Hanzeplein 1, 9713 GZ Groningen, The Netherlands

## Abstract

**Introduction:**

Lupus nephritis (LN) is a severe and frequent manifestation of systemic lupus erythematosus (SLE). Early detection of initial renal manifestations and relapses during follow-up is pivotal to prevent loss of renal function. Apart from renal biopsies, current urinary and serological diagnostic tests fail to accurately demonstrate the presence of active LN. Previously, we demonstrated that effector memory T-cells (CD45RO^+^CCR7^-^;T_EM_) migrate into the urine during active LN. The objective of this study was to assess the diagnostic value of urinary T-cells in comparison with traditional markers of active LN.

**Methods:**

T-cells in the urine during active LN and remission were investigated. Twenty-two, in most cases biopsy-proven, active LN patients and 24 SLE patients without active LN were enrolled and serial measurements were performed in 16 patients.

**Results:**

Analysis of the urinary sediment in active renal disease showed an increased number of CD8^+ ^T-cells and absence of these cells during remission. Enumerating T-cell counts in LN patients with a history of renal involvement was a superior marker of active LN in comparison to traditional markers, such as proteinuria and s-creatinine.

**Conclusions:**

In conclusion, urinary T-cells, in particular CD8^+ ^T cells, are a promising marker to assess renal activity in LN patients, in particular in those with prior renal involvement.

## Introduction

Systemic lupus erythematosus (SLE) is an autoimmune disease characterized by manifestations in multiple organs. Inflammation of the kidneys, in particular, is associated with an unfavorable prognosis [1,2]. Although the precise pathogenesis of lupus nephritis (LN) has not been fully elucidated, kidney infiltrating T-cells seem to contribute to the inflammatory pathology of LN [3].

Evaluation for LN includes dipstick and urine sediment analysis, urinary protein and creatinine excretion, determination of serum creatinine and assessment of serological markers, such as anti-dsDNA antibody titers and C3 and C4 levels [4]. The combination of these markers is a powerful measure for the detection of active renal manifestations of SLE. However, in clinical practice, traditional clinical markers for renal involvement, such as proteinuria, not always discriminate between active and inactive disease, in particular in patients with a recent history of LN [5]. In these patients persistent proteinuria often limits the information of this test to detect renal flares or remission. This is due to the fact that proteinuria might reflect both glomerular damage and renal activity. For these patients, strict guidelines defining renal flares based on laboratory information are lacking [6]. Therefore, renal biopsies are crucial and are still the gold standard to assess renal disease and to define the histo-pathologic class of LN [7]. This invasive approach is associated with a risk of bleeding and repeated renal biopsies are not always applicable in daily clinical practice in patients with SLE.

Thus, novel non-invasive urinary markers seem to be an attractive goal to detect renal flares in LN. Several studies demonstrated the presence of mononuclear cells in urine of patients with active IgA nephropathy, LN and Wegener's granulomatosis [8-10]. Recently, we reported an increase of urinary T_EM_-cells (CD45RO^+^CCR7^-^) in patients with active LN [11]. Remarkably, these cells were almost absent in healthy controls and random lupus patients without active LN. These data suggest that measuring urinary T-cells might also be helpful in discriminating active LN versus patients with a recent history of LN but without current active renal disease. Therefore, in a serial cohort of LN patients traditional clinical markers and urinary T-cell counts were analyzed at the time of active and inactive renal disease to evaluate the significance of urinary T-cell measurements for assessing renal activity. The present data suggest that measuring urinary T-cells, in particular CD8^+ ^T cells, might be an additional diagnostic tool to determine renal disease activity, particularly in patients with a recent history of lupus nephritis.

## Materials and methods

### Study population

A total of 46 SLE patients fulfilling at least four of the American College of Rheumatology revised criteria for SLE were enrolled in this study [12]. Twenty-four patients, including 14 patients without a history of renal involvement, have been described before [11]. Twenty-two patients were enrolled with active LN (Table 1). Disease activity was assessed by SLEDAI (SLE Disease Activity Index) and active SLE was defined as a SLEDAI score >4. Mean disease activity for active LN patients was 13 ± 3 (Table 1). Median (range) anti-dsDNA titers were 230 (3 to 1,000 E/ml), median C3 and C4 were 0.55 g/l (0.05 to 1.03) and 0.14 g/l (0.04 to 0.30). Twenty-four 24 hour-proteinuria was 2.8 ± 3.3 g/l among active LN patients. Active LN was defined by at least two of the following items: (i) new onset proteinuria >0.5 g/24 h, (ii) an active urinary sediment representing glomerular injury and (iii) a renal biopsy providing evidence of active lupus nephritis (*n *= 21) (Table 1). According to the International Society of Nephrology (ISN) classification, histopathology showed class II (*n *= 1), class III (*n *= 9), class III/V (*n *= 1), class IV (*n *= 10) or unclassified LN (*n *= 1) in the present cohort. The mean activity index (AI) according to Austin was 4.6 ± 2.6. All active LN patients fulfilled the renal BILAG-2004 category A criteria and, subsequently, received an escalation of immunosuppressive treatment [13]. Sixteen patients (class II (*n *= 1), class III (*n *= 7), class IV (*n *= 8)) were analyzed twice, both during active renal disease and inactive renal disease. The time between the visits was 14 ± 4 months. Inactive renal disease was defined as renal BILAG-2004 category B or C. Thus, in these patients renal function was stable or improving and there was no need to escalate or change maintenance therapy. Medication of patients with active and inactive LN is shown in Table 2. Informed written consent was obtained from patients after approval of the study by the Medical Ethics Committee of the University Medical Center Groningen. The study was conducted according to the ethical guidelines of our institution and the Declaration of Helsinki.

**Table 1:** Baseline characteristics of LN patients included in the study (*n *= 46)

	Active LN	Without active LN	
		
		History of LN	No history of LN
Total number	22	10	14
Women/men	19/3	7/3	13/1
Age (years, mean ± SD)	35 ± 10^*^	46 ± 14	37 ± 10
SLEDAI (mean ± SD)	13 ± 3 ^**, ##^	3 ± 2	3 ± 3
Anti-dsDNA, E/ml (median, range)	230 (3 to 1,000)^*, ##^	5 (3 to 432)	81 (3 to 1,000)
C3 g/l (median, range)	0.54 (0.05 to 1.03)^**, ##^	1.13 (0.54 to 1.48)	0.99 (0.31 to 1.43)
C4 g/l (median, range)	0.08 (0.03 to 0.48)^*^	0.23 (0.04 to 0.30)^$^	0.12 (0.05 to 0.24)
			
Previous biopsy proven LN (yes/no)	9/13	10/0	0/14
Serum creatinine µmol/l	94 ± 78^#^	80 ± 26	62 ± 13
			
Urinary analysis			
Proteinuria g/24 h (mean ± SD)	2.8 ± 3.2^**, ##^	0.3 ± 0.3^$,$^	0.0 ± 0.1
Dysmorphic erythrocytes in %	36 %	0.%	0.%
CD4^+^T-cells/ml (median, range)	128 (0 to 1,250)^**, ##^	9 (0 to 63)	0 (0 to 136)
CD8^+^T-cells/ml (median, range)	177 (0 to 1,388)^**, ##^	13 (0 to 118)	17 (0 to 92)

The patients were sub-grouped into biopsy proven LN (*n *= 22) and patients without active LN (*n *= 24). The patients without active LN were further sub-grouped into patients with biopsy proven LN in the past (*n *= 10) and patients without renal involvement (*n *= 14). Significant differences between patients with active LN and patients with biopsy proven LN in the past without current activity are indicated (*P *<0.05 = *, *P *<0.005 = **). Significant differences between active LN and patients without renal involvement are indicated (*P *<0.05 =^#^, *P *<0.005 =^##^). Significant differences between patients with biopsy proven LN in the past without current activity and patients without renal involvement are indicated (*P *<0.05 =^$^, *P *<0.005 =^$$^).

**Table 2:** Use of immunosupressive drugs in patients with active lupus nephritis (LN) and inactive LN patients

	Active LN	Inactive LN	*P-value*
Total number	22	24	
Treatment, n			
None	8	3	0.038
Glucocorticoids	10	13	
*Median dose (range), dose (mg/day)*	5 (2.5 to 15)	5 (1.25 to 10)	
Immunosupressive/immunomodulating, n			
Hydroxychloroquine	9	8	
*Median dose (range), users (mg/day) *	400 (200 to 600)	400 (200 to 600)	
Methotrexate	1	2	
*Median dose (range), users (mg/week) *	7.5	13.75 (12.5 to 15.0)	
Azathioprine	4	8	
*Median dose (range), users (mg/day) *	100 (75 to 150)	112.5 (75 to 150)	
MMF	1	3	
*Median dose (range), users (mg/day) *	2,000	3,000 (2,000 to 3,000)	

### Materials

EDTA-blood and fresh urine samples were collected from patients. Urine samples from patients which were nitrite positive on a dip stick test or with proof of bacterial contamination in the sediment were excluded.

Percentages and absolute counts of CD4^+ ^and CD8^+ ^T-cells were assessed immediately after sampling by four-color flow cytometry in blood and urine samples. Paraffin-embedded sections of renal biopsy specimens obtained from 22 patients were included in the present study.

### Antibodies

The following antibodies were used in flow cytometry: phycoerythrin (PE)-conjugated anti-CCR7 (clone 3D12), fluorescein (FITC)-conjugated anti-CD45RO (clone UCHL-1), peridin-chlorophyll (PerCP)-conjugated anti-CD4 (clone SK3), allophycocyanin (APC)-conjugated anti-CD3 (clone UCHT1), MultiTEST™ four-color antibodies (CD3-FITC, CD8-PE, CD45-PerCP and CD4-APC), and isotype matched control antibodies of irrelevant specificity. All were purchased from Becton-Dickinson ((BD), Amsterdam, The Netherlands).

### Sample preparation and flow cytometry

Immediately after voiding, urine was diluted 1:1 with cold phosphate-buffered saline (PBS) and processed as described before. Briefly, isolated mononuclear cells were resuspended in wash-buffer (1% BSA in PBS) and mixed with appropriate concentrations of anti-CD45RO-FITC, anti-CCR7-PE, anti-CD4-PerCP and anti-CD3-APC for 15 minutes at room temperature in the dark. In parallel, blood samples were labeled with the aforementioned monoclonal antibodies. Afterwards, cells were successively treated with 2 ml diluted FACS lysing solution (BD, Amsterdam, The Netherlands) for 10 minutes and samples were washed twice in wash-buffer and immediately analyzed by flow cytometry. Four-color staining was analyzed on FACS-Calibur (BD, Amsterdam, The Netherlands) and data were collected for 10^5 ^events for each sample and plotted using Win-List software package (Verity Software House Inc., Topsham, ME, USA). Positively and negatively stained populations were calculated by quadrant dot-plot analysis, as determined by the isotype controls.

### Quantification of effector memory T-cells

T-cells were quantified in urine using TruCOUNT™ Tubes (BD, Amsterdam, The Netherlands). In brief, 20 µl of MultiTEST™ four-color antibodies (CD3 FITC, CD8 PE, CD45 PerCP and CD4 APC) and 50 µl of sample (urine or blood) were added to bead-containing TruCOUNT™ tubes. The cell suspension was processed and analyzed as described elsewhere. Afterwards, the absolute counts for T_EM _cells in 1 ml urine were calculated as described before [8].

### Analysis and scoring of renal biopsies

Biopsies taken at the time of analysis of blood and urine samples were processed. All biopsies were reviewed and classified by an experienced nephropathologist (MvD) according to the revised criteria for LN. The AI and chronicity index (CI) were calculated for each specimen with maximum scores of 24 for the AI and 12 for the CI. For this study, methenaminesilver-stained slides (with HE-counterstaining), H&E and periodic acid-Schiff (PAS) stained slides were used.

The assessment was completed by determining the ISN/RPS2003 classification and activity and chronicity indices for LN [14]. For these aspects of the assessment, the definitions of the classification systems and the activity and chronicity indices were used.

### Immunohistochemistry staining

All specimens were fixed in 10% neutral buffered formalin and paraffin embedded. Five-micrometer-thick sections were deparaffinized in xylene and rehydrated in a series of different concentrations of ethanol. EDTA buffer, pH 8.2, for heat-induced epitope retrieval was applied for 1 h, followed by neutralization of endogenous peroxidase with 0.3% H_2_O_2_. Incubation with a monoclonal mouse anti-human CD8 (DAKO, Glostrup, Denmark) was performed. Next, sections were washed and incubated with a HRP-conjugated secondary antibody (Envison™, DAKO, Glostrup, Denmark) for 30 minutes at room temperature. A DAB substrate was used for visualization. Washing with PBS was performed after each incubation step. CD4 (Roche/Ventana Medical Systems Inc., Oro Valley, AZ, USA)) was performed in the Benchmark Ultra (Roche/Ventana) with citrate buffer heat inducted antigen retrieval and detected with the *ultra*View Universal DAB detection Kit (Roche/Ventana). Finally, the slides were counterstained with hematoxylin and mounted with Kaiser's glycerine gelatin (Merck, Darmstadt, Germany).

Cells were separately counted for the interstitium and glomeruli. Cells with positive staining for CD8 and CD4 were counted per high powerfield (40x magnification). The average value was calculated for each biopsy.

### Statistical analysis

Based on our prior findings regarding urinary CD8^+ ^T-cells in active (mean 287 ± 220 cells/ml) versus inactive LN patients (mean 22 ± 28 cells/ml) [11], we conducted a power analysis using G*Power 3.1.5. A total sample size of *n *= 18 achieves 90% power to detect this difference of CD8^+ ^T-cells between the mean of both groups (effect size: 1.69) using a two-sided hypothesis test with a significance level (alpha) of 0.05.

Results were presented as median (range) or mean ± SD and the nonparametric Mann-Whitney U-test was used for comparison of values between groups. Paired samples were tested using the Wilcoxon signed rank test. Correlation with disease activity was assessed using Spearman's rank correlation coefficient. Receiver operator curve (ROC) analysis was performed for urinary T-cells and proteinuria to evaluate the discriminative power between patients with active vs. inactive LN. All analyses were performed using GraphPad Prism 5.0 (GraphPad Software Inc., La Jolla, CA, USA). *P*-values less than 0.05 were regarded as statistically significant.

## Results

### CD8^+ ^T-cell count discriminates between active and inactive LN

We detected T-cells in the urine in 21 active LN patients out of the 22. Only one active LN patient presented without urinary T-cells, which means a sensitivity of 95%. The median (range) count of urinary CD4^+ ^T-cells was significantly increased in active LN patients in comparison to inactive LN patients (128 (0 to 1,250 cells/ml) vs. 5 (0 to 136 cells/ml), respectively (*P *<0.0001)) (Table 1). The median (range) count of urinary CD8^+ ^T-cells was also significantly increased in active LN patients in comparison to inactive LN patients (177 (0 to 1,388 cells/ml) vs. 14 (0 to 118 cells/ml), respectively (*P *<0.0001)). The median urinary CD8^+^/CD4^+ ^ratio was 1.7, suggesting that predominantly CD8^+ ^T-cells are migrating into the kidney.

ROC analysis revealed that the urinary CD4^+ ^and CD8^+ ^T-cell count discriminates between active and inactive LN (area under the curve (AUC) 0.92 and 0.93, respectively). Urinary T-cell counts were somewhat weaker indicators for active renal disease than proteinuria (AUC 0.97) but superior to s-creatinine (AUC 0.60). There was no significant correlation between urinary CD4^+ ^or CD8^+ ^T-cell counts and AI or CI, respectively.

### Decrease of urinary T-cell counts after induction of remission

**Figure 1:**
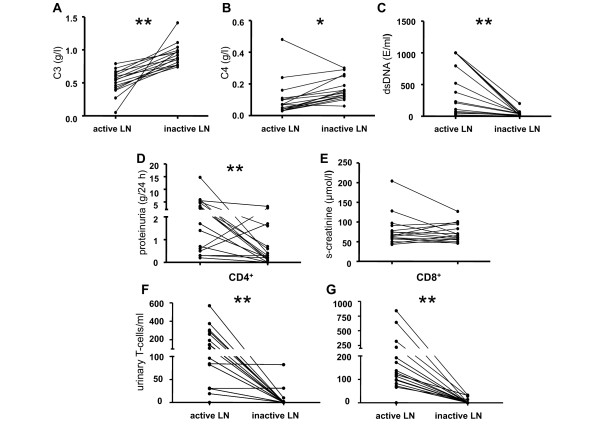
**Laboratory data of serially analyzed LN patients (*n *= 16) during active and inactive LN**. Intra-individual comparison between levels of C3, C4, anti-dsDNA titres, proteinuria, s-creatinine, urinary CD4^+ ^and CD8^+ ^T-cell counts in lupus nephritis (LN) patients (*n *= 16) during active and inactive LN is shown in sections **A-G**. The time interval between these two assessments was 14 ± 4 months. *P*-values were calculated using the nonparametric Wilcoxon signed rank test. Significant differences are indicated (*P *<0.05 = *, *P *<0.005 = **).

Urine specimens of 16 patients were analyzed at the time of active renal disease and remission (Δ *t = *14 ± 4 months) to assess intra-individual changes in urinary T-cell counts. Fifteen of these patients were active at baseline and treated with induction therapy for LN. One patient was in renal remission at baseline and had a biopsy proven relapse during follow-up. Urinary CD4^+ ^T-cell counts decreased significantly from 125 (0 to 569 cells/ml) to 0 (0 to 82 cells/ml, *P *= 0.001, Figure 1) from active renal disease into remission. CD8^+ ^T-cells decreased from 124 (0 to 841 cells/ml) to 0 (0 to 33 cells/ml, *P *<0.0001). SLEDAI scores decreased significantly from 13 ± 3 to 3 ± 2 (*P *<0.0001).

In accordance with changes in disease activity anti-dsDNA titers decreased significantly from 230 (3 to 1,000 E/ml) to 29 (3 to 200 E/ml, *P *= 0.0006). C3 complement levels increased from 0.55 (0.05 to 0.79 g/l) during active LN to 0.90 (0.74 to 1.41 g/l, *P *= 0.0005) during quiescent renal disease. C4 complement levels increased from 0.07 (0.03 to 0.48 g/l) during active LN to 0.15 (0.06 to 0.30 g/l, *P *= 0.008) during quiescent renal disease. Serum creatinine levels remained stable at 79 ± 40 µmol/l in active LN patients as compared to 71 ± 23 µmol/l in inactive LN patients. The 24-h urinary protein excretion decreased significantly from 3.1 ± 3.6 g/l to 0.7 ± 1.0 g/l (*P *= 0.005) (Figure 1A-E). Importantly, although all parameters (except serum creatinine) significantly changed when patients turned from active into inactive LN, there remained considerable overlap. For example, 5 out of 16 patients with inactive lupus nephritis had 24-h urinary protein excretion levels of >0.5 g/l. In contrast, urinary CD8^+ ^T-cell counts in all patients with inactive LN could be discriminated from those during active LN as no overlap in urinary CD8^+ ^T-cells occurred.

### T-cell count discriminates between active and inactive LN, independent of a history of LN

To assess the additional value of urinary T-cell counts to discriminate active from inactive LN, in particular in patients with a history of biopsy proven LN, we subdivided the cohort of patients without active LN in two groups, one group without renal involvement (*n *= 14) and one group with patients with a history of LN (*n *= 10). Proteinuria was significantly increased in patients with a history of LN as compared to patients without LN (0.3 ± 0.3 g/24 h vs. 0.0 ± 0.1 g/24 h, *P *= 0.002). There was no difference in serum creatinine levels between both groups.

**Figure 2:**
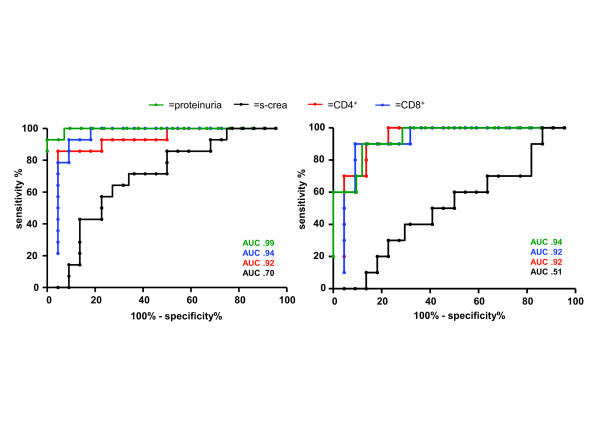
**Receiver operator curve (ROC) analysis for proteinuria (g/24 h), serum creatinine, urinary CD4^+ ^and CD8^+ ^T-cells comparing SLE patients with active renal disease (*n *= 22) vs**. patients without renal involvement (*n *= 14) (**A**). The same parameters are shown comparing patients with active LN (*n *= 22) vs. patients with a history of LN but without current renal activity (**B**). The area under the curve (AUC) is shown for all parameters.

ROC analysis (Figure 2A) shows that proteinuria is an excellent marker to discriminate patients with active LN from patients without renal involvement (AUC 0.99). The discriminative power was somewhat lower in patients with active LN versus inactive LN patients (AUC 0.94) (Figure 2B). In contrast, in this cohort the discriminative power of urinary T-cell counts was comparable with respect to urinary CD4^+ ^T-cells (AUC 0.92) and CD8^+ ^T-cells (AUC 0.92).

### T-cells in renal biopsies

To determine the presence and localization of CD8^+ ^T-cells and CD4^+ ^T-cells in renal biopsies, immunohistochemistry staining with anti-CD8 and anti-CD4 was performed.

T-cells were present in all except one renal biopsy investigated. The mean amount of CD8^+ ^T-cells was 5.0 ± 4.3 cells/high power field. This was significantly higher than the mean amount of CD4^+^T-cells (2.4 ± 2.8 cells/high power field; *P *= 0.006). Urinary CD8^+ ^T-cells did not correlate significantly with the amount of kidney infiltrating CD8^+ ^T-cells (r = 0.28, *P *= 0.29). The number of urinary CD4^+ ^T-cells correlated significantly with the number of CD4^+ ^T-cells/high power field in these renal biopsies (r = 0.53, *P *= 0.04). There was no significant correlation between T-cell numbers in the urine and renal AI (data not shown).

## Discussion

The present study, in accordance with our preliminary data, demonstrates that urinary effector T-cells are increased during flares of LN. Urinary T-cells with an effector-memory phenotype can be detected in almost all active LN patients. Several studies demonstrated an increased number of kidney infiltrating T-cells in LN patients [15-18]. In these reports the authors investigated chemokine receptor expression of T-cells to explain the underlying mechanism of T-cell recruitment. They found, in particular, a selective accumulation of CCR4^+ ^and CXCR3^+ ^T-cells accompanied by a decrease of these subsets in the peripheral blood [15,17]. A study by Enghard *et al*. showed that CD4^+^CXCR3^+ ^T-cells are infiltrating into the inflamed kidneys and can be detected in the urine during acute renal flares [17]. In agreement with our data, CD4^+ ^T-cells were increased in active renal disease. However, our findings show that CD8^+ ^T-cell counts seem to differentiate more accurately between active and inactive renal disease. Besides, the aforementioned studies did not include serial measurements in representative cohorts of SLE patients.

In the context of the clinical course of LN, T-cell recruitment and migration might be restricted to active disease. In order to demonstrate that urinary T-cells are specific for renal activity in SLE we assessed T-cell counts in a cohort with active LN and a cohort of patients without active LN, including patients with a history of LN. A significantly increased number of urinary T-cells was detectable during active LN. This was especially true for the CD8^+ ^T-cell count, as no overlap in CD8^+ ^T-cell counts was observed between active and inactive LN patients. This seems to reflect the histopathological findings at least to a certain extent in active renal lupus nephritis, which is supported by the presence of CD8^+ ^and an intra-renal CD4^+^/ CD8^+ ^ratio <1. Besides, we have to admit that the specificity and power of this new tool is limited by the lack of kidney biopsies from inactive lupus nephritis patients due to ethical reasons.

The present data suggest that measuring urinary T-cells could serve as an additional diagnostic marker for LN. ROC analysis was performed for urinary T-cells and proteinuria to evaluate their power to discriminate between active LN patients and inactive LN. This analysis showed that 24 h proteinuria (AUC 0.97) is still superior to distinguish between active LN patients and patients without active LN (Figure 2). However, the presented subanalysis reveals also that the discriminative power is related to renal involvement. Proteinuria discriminates sharply between active LN patients and patients without renal involvement (AUC 0.99). However, the diagnostic value decreases in patients with a history of LN (AUC 0.94).

More difficulties exist in assessing renal activity in patients with recently active LN. Therefore, we analyzed traditional renal markers in our follow-up cohort. In these patients, persistent proteinuria is a frequent finding and a predictor of chronic kidney disease [19]. Although a significant decrease of proteinuria could be observed in this cohort, almost 30% of patients were still excreting protein at the time of remission (≥0.5 g/l in 24 h). This limits in these patients the diagnostic value of proteinuria for the early detection of renal flares whereas CD8^+ ^T-cell counts sharply discriminates between these groups (*P *<0.0001).

Despite the suboptimal predictive value of proteinuria for renal flares, the combined evaluation of urinary protein excretion and microscopic urine analysis is still the routine standard to assess renal disease activity in LN [20]. In some cases, assessment of urinary protein excretion is not conclusive, especially in patients with longstanding renal involvement and persistent proteinuria. The presence of an active urinary sediment, in particular the presence of dysmorphic erythrocytes, then is a necessary requirement. However, in our experience, the supplementary information of microscopic urine analysis is somewhat limited. The quality of the urinary specimens is often inappropriate and microscopic urine analysis revealed only in a restricted number (8 out of 22) of biopsy proven active LN patients an active sediment. Additionally, clear guidelines and definitions of active LN in patients with a history of renal injury are lacking [21].

## Conclusions

In conclusion, the present study strengthens our preliminary observation that urinary effector T-cells are increased during renal flares of LN. Moreover, these data suggest an additional diagnostic role of measuring urinary T-cells. This analysis will be especially helpful in LN patients with a recent history of LN. This relates to the fact that persistent proteinuria is a frequent observation which limits the use of this marker for the judgement of renal activity. Measuring urinary CD8^+ ^T-cells helps to discriminate between active and inactive LN. To introduce this test into routine diagnostics, further studies are needed to confirm these results in a larger cohort of biopsy-proven LN patients.

## Abbreviations

AI: activity index; APC: Allophycocyanin; AUC: area under the curve; BD: Becton-Dickinson; BILAG-2004: British Isles Lupus Assessment Group-2004; BSA: bovine serum albumin; CI: chronicity index; EDTA: ethylenediaminetetraacetic acid; FCS: fetal calf serum; FITC: fluorescein isothiocyanate; ISN: International Society of Nephrology; LN: lupus nephritis; PAS: periodic acid-Schiff; PBS: phosphate buffered saline; PE: Phycoerythrin; PerCP: peridin chlorophyll protein; ROC: receiver operator curve; SLE: systemic lupus erythematosus; SLEDAI: systemic lupus erythematosus disease activity index; T_EM_: effector memory T-cells

## Competing interests

The authors declare that they have no competing interests.

## Authors' contributions

All authors contributed to the design, acquisition and interpretation of data. SD and WHA performed the flow cytometry and *in vitro *experiments and drafted the manuscript. SD performed the statistical analysis and contributed to the interpretation of the data. MCRFvD and SD assessed the renal specimens. PCL contributed to concept and design, and interpretation of data. SA contributed to the statistical analysis and the interpretation of the data. CGMK contributed to concept and design, interpretation of data and the revision of the article. MB contributed to concept and design, inclusion of SLE patients, interpretation of data and the drafting of the article. All authors read and approved the final manuscript.
